# Trends in annual drug expenditure – a 16 year perspective of a public healthcare maintenance organization

**DOI:** 10.1186/s13584-016-0096-1

**Published:** 2016-09-15

**Authors:** Yossef Lomnicky, Daniel Kurnik, Ronen Loebstein, Itzhak Katzir, Janet Vesterman-Landes, Nava Siegelmann-Danieli

**Affiliations:** 1 Department of Pharmaceutics and Clinical Pharmacology, Maccabi Healthcare Services, Tel-Aviv, Israel; 2 Department of Professional Medicine, Maccabi Healthcare Services, Tel-Aviv, Israel; 3 Health Division, Maccabi Healthcare Services, HaMered St. 27 Tel Aviv, Israel; 4 Sackler School of Medicine, Tel Aviv University, Tel Aviv, Israel; 5 Rappaport Faculty of Medicine, Technion-Israel Institute of Technology, Haifa, Israel

**Keywords:** Drug expenditure, Cancer agents, Health Maintenance Organization

## Abstract

**Background:**

Modern drug therapy accounts for a major share of health expenditure and challenges public provider resources. The objective of our study was to compare drug expenditure trends for ten major drug classes over 16 years at Maccabi Healthcare Services (MHS), the 2^nd^ largest healthcare organization in Israel.

**Methods:**

A retrospective analysis of drug expenditure per HMO beneficiary between the years 1998–2014. Trends in annual mean drug expenditures per MHS member were compared among 10 major drug classes.

**Results:**

Average annual drug expenditure per beneficiary increased during the study period from 429.56 to 474.32 in 2014 (10.4 %). Ten drug classes accounted for 58.0 % and 77.8 % of total drug cost in 1998 and 2014, respectively. The overall distribution of drug expenditure among drug classes differed significantly between 1998 and 2014 (*p* < 0.001), mainly due to the increase in expenditure for cancer drugs, from 6.8 % of total drug cost to 30.3 %. In contrast, expenditures for cardiovascular drugs decreased during the same period from 16.0 to 2.7 %. Moreover, the median annual increase in net drug costs per HMO member during 1998–2014 was largest for cancer drugs (NIS 6.18/year; IQR, 1.70–9.92/year), about two-fold that of immunosuppressants, the second fastest growing drug class (NIS 2.81; IQR, 0.58–7.43/year).

**Conclusions:**

The continuous rise in anti-cancer drug expenditure puts a substantial burden on the medication budgets of public health organizations. Coordinated measures involving policy makers, physicians, and pharmaceutical companies will be required for efficient cost containment.

## Background

Oncology drug cost, amounting to an estimated $40 billion per year worldwide, is a concerning issue in public health economic discussions [1]. Medicare spending on Part B drugs — a category dominated by drugs used to treat cancer — increased from $3 billion in 1997 to $11 billion in 2004 (a 267 % increase), compared with a rise in overall Medicare spending from $210 billion to $309 billion (an increase of 47 %) during the same period [2]. While 15 years ago the most expensive cancer drug was paclitaxel (TAXOL®, Bristol-Myers Squibb) at a monthly cost of approximately US$ 2,500 per patient in the USA, some recently approved anti-cancer drugs are much more expensive (http://www.ascopost.com/issues/february-1,-2013/cost-of-cancer-drugs-what-price-for-what-benefit.aspx). Trends in oncology drug expenditures, especially in comparison with drugs for non-oncology indications, have been only partially characterized. When disclosed by health organizations, drug expenditure often includes additional fees associated with the specific medical condition such as hospital admission bills, surgeries and procedures, ambulatory care cost and supportive therapy, all of which may vary widely among countries [3–5].

Israel has national health insurance with universal health coverage, i.e. every citizen is insured by one of four health maintenance organizations (HMOs). The budget of each HMO is allocated by the government based on the number of beneficiaries. Medications and health technologies and their precise indications covered by the HMOs are determined by the Ministry of Health and summarized in the “Health basket”. The government decides on the annual growth rate of the health basket’s budget, and once a year a professional committee prioritizes the addition of new medications or technologies and their indications.

Maccabi Healthcare Services (MHS) is the second largest Israeli health organization, insuring about 2 million beneficiaries in 2014 nationwide. Its health benefits include medications and technologies as specified by the Israeli National Health Basket. Additionally, MHS provides new health technologies, including medications with evidence-base technologies prior to their inclusion in the National Health Basket. Our objective was to compare trends in drug expenditure at a large modern HMO over a 16-year- period, in major drug classes representing therapies for diverse acute and chronic medical conditions.

## Methods

### Data extraction

All data regarding drug purchases (quantities and prices) were retrieved from the MHS computerized database. This database includes information on all medications dispensed to MHS beneficiaries starting January 1998 up to end of 2014, classified into 56 pharmacological groups. We included in our analyses the 10 major drug classes that accounted for about three quarters of the total drug expenditure in 2014: Immunosuppressants, anti-cancer drugs, immuno-stimulants, antiviral drugs, anti-diabetic drugs, cardiovascular drugs, anticoagulants, enzyme replacement agents, lipid-lowering drugs, and antibiotics.

### Data analysis and statistics

Data on drug expenditure were expressed in New Israeli Shekels (NIS) and matched to the Israeli 2014 Cost of Health Index. For the years 1998–2014 (observation period), we calculated the expenses for each drug category per HMO member, thus accounting for the growing number of HMO members during this period. Changes in annual drug expenses per HMO member were not normally distributed, and were therefore expressed as medians and interquartile ranges, with non-parametric tests (Kruskal-Wallis) used for comparison among different drug categories. Additionally, we compared the distribution of expenditures for the 10 drug classes as proportions of total drug expenditures at the beginning (1998) and the end of the observation period (2014) using χ^2^-test. For all statistical analyses, P-values <0.05 were considered significant. Statistical analyses were performed using SPSS statistical software (SPSS v.22, IBM® SPSS® Inc., Chicago, IL). The study was approved by the MHS institutional review board.

## Results

### Global drug expenditure

During the study period, the number of MHS beneficiaries increased by 88.6 %, from 1.141 to 2.151 million. The total annual drug purchase expenditures increased by 108.2 %, from NIS 489.9 million to NIS 1,020.2 million. Accordingly, the average annual drug expenditure per beneficiary increased only slightly, from NIS 429.56 in 1998 to 474.32 in 2014 (a rise of 10.4 %). The ten major pharmacological groups accounted for 58.0 % and 77.8 % of total drug cost in 1998 and 2014, respectively.

### Trends in drug expenditure by drug category

**Fig. 1:**
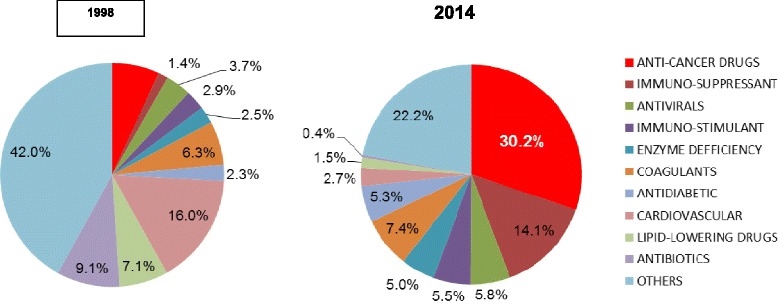
Distribution of drug expenditure for ten major drug classes per HMO member in 1998 and 2014. The overall distribution differed significantly between 1998 and 2014 (*P* < 0.001)

Costs per beneficiary in 1998 were dominated by cardiovascular medications (16.0 % of total cost), followed by antibiotics (9.1 %) and lipid-lowering drugs (7.1 %). Costs of cancer agents accounted for 6.8 % of drug expenditure in 1998, but rose steadily to become the largest expenditure for a single drug class starting in 2002. By 2014, antineoplastic drugs accounted for 30.2 % of total drug expenditure, while expenditure for some other drug classes (e.g., cardiovascular and lipid-lowering drugs and antibiotics) decreased constantly. Accordingly, the overall distribution of drug expenditures for different drug classes was significantly different when comparing the beginning (1998) with the end (2014) of the observation period (*P* < 0.001; Fig. 1).

**Fig. 2:**
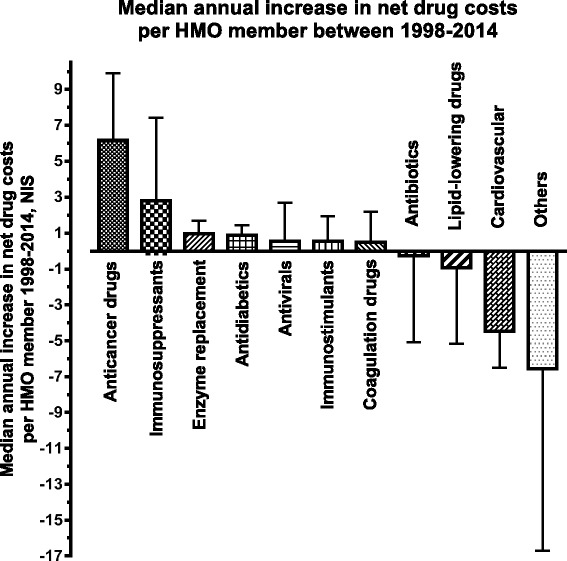
Median annual change in net drug expenses per HMO member for different drug classes between 1998 and 2014. Changes in expenditure were significantly different among drug classes (*P* < 0.001). Bars denote the median, error bars the 75 % percentile

Differing trends in drug expenses during the observation period were also evident when examining the annual changes in drug expenditures. The median annual increase in drug expenses per beneficiary during the observation period differed significantly among drug classes (overall Kruskal-Wallis *P* < 0.001; Fig. 2).

For cancer drugs, expenses increased by a median of NIS 6.18 (IQR, 1.70–9.92) per HMO beneficiary each year, an annual increase that was more than two-fold higher compared to that of immunosuppressants (NIS 2.81; IQR, 0.58–7.43), but the difference was not statistically significant (Mann-Whitney test for comparison between these two drug classes, *P* = 0.13).

## Discussion

Our study demonstrates that expenditures for cancer agents have been rising constantly over the last decade, offsetting savings for the majority of medications used to treat other acute and chronic medical conditions. In fact, by the end of study period approximately a third of all MHS drug expenditure was accounted for by anti-cancer drugs.

In this study, we did not attempt to identify the contribution of specific reasons for the increasing expenditure for oncological therapies, e.g., increasing drug prices, increasingly complex (and therefore more expensive) treatment regimens, or an increasing proportion of patients treated, either because of changes in cancer prevalence/detection or changes in eligibility criteria for cancer. However, the overall age-adjusted rates of invasive tumors in males and females have changed only minimally during the study period [6]. Moreover, all therapies included in the health basket follow internationally accepted evidence-based guidelines.

One likely reason for the sharp increase in expenditures for cancer agents is the increasing approval of expensive biological and targeted therapies in this field (e.g. monoclonal antibodies and modern tyrosine kinase inhibitors) [7]. This trend is likely to continue with the inclusion of costly checkpoint inhibitors (Programmed Death-1 [PD-1] inhibitors) in the national health basket in 2015 (i.e., after the end of the study period). Biologic drugs also contributed to the increased drug expenditure for autoimmune conditions in our study since 2006, paralleling their introduction for treatment of inflammatory bowel diseases, rheumatic diseases, and other autoimmune disorders, and challenged by health organizations’ difficulties to incorporate biosimilars.

Rising costs of cancer drugs should be viewed with concern in light of the fact that cancer incidence is expected to rise worldwide, from 10.4 million newly diagnosed patients annually in 2000–25 million in 2030 [8], with more than 70 % of cases occurring in low- and middle-income countries [9].

**Fig. 3:**
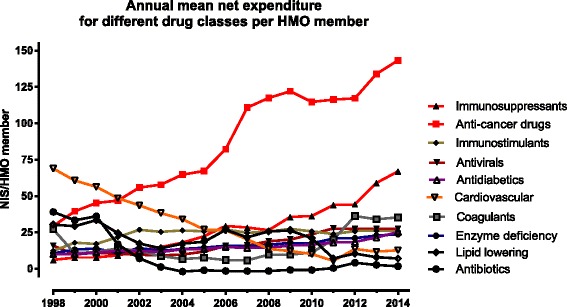
Mean annual net expenditure per HMO member for the major drug classes. The listed drug classes comprised together 77.8 % of the total drug expenditure per MHS member in 2014

The increase in ***total*** drug expenditure per beneficiary in our study (a rise of 10.4 % over 16 years) was modest compared to other developed countries, e.g., 5.5 % and 4 % in Western European countries and Japan over shorter time periods, respectively [4, 5]. This difference reflects extensive generic drug use in Israel for numerous medical conditions compared with other Western countries [4]. In our study, the major savings in expenditure for antibiotics, cardiovascular drugs and lipid lowering agents were most noticeable after 2001 (Fig. 3), coinciding with the HMO-wide implementation of cost containment methods including preferred drug lists, generic substitution, pre-authorization of selected expensive drugs, and advanced methods to assess and control drug prescription and dispensing.

The allocation of limited public resources to various diseases has to be judged objectively in view of the expected benefit to patients. During the years 1970–2000, the life expectancy of U.S. Americans increased on average by 6 years; only 6 months were attributed to antineoplastic therapies, while over 4 years were attributed to pharmacological interventions for cardiovascular diseases and metabolic conditions [10]. Many drug treatments for cardiovascular diseases and metabolic conditions are available as generics, and “me-too” or “glorified-me-too” drugs were released, increasing competition and diminishing overall costs for these pharmaceutical groups. Notwithstanding, public awareness of the needs of cancer patients in Western countries, where malignancy is the leading cause of death, increases the pressure on regulators to approve new and expensive cancer agents, sometimes with only limited evidence of cost-effectiveness [11, 12]. Moreover, data on post-marketing effectiveness, including the effect on mortality in cancer patients, is sparse, precluding the assessment of real-life public benefit.

Our study has several limitations. Firstly, MHS ensures a relatively young population [16 % are ≥55 years of age, as compared to 19.1 % in the general Israeli population [13]. Our study may therefore underestimate the expenditure for antineoplastic agents in aging populations. As mentioned above, we normalized expenditure per HMO beneficiary and not per patient treated, which greatly underestimates the real rise in drug expenditure per cancer patient. However, the focus of this study was the changing burden of drug expenditure across different drug classes on an HMO’s medication budget, and not expenditures per patient for individual patient groups. Our approach better captures the true impact of expenditures for a pharmacological class on the budget, since it reflects both drug costs per patient and the prevalence of the disease group. For instance, drug expenditure for a patient with Gaucher disease requiring enzyme replacement therapy is higher than for the average oncological patient, yet the burden of Gaucher patients on the medication budget is lower (5 % of total expenditures) due to the low number of Gaucher patients compared to that of oncological patients (30 % of total expenditures). We also did not consider expenditure for drugs used in oncology supportive care, such as agents to control chemotherapy-induced nausea and vomiting, narcotics and other analgesics, and growth factors (e.g. G-CSF), and our data therefore underestimate the real malignancy-related drug expenditure for cancer patients. A few oncological medications also have specific non-oncological indications, and are covered by the health basket according to specified criteria as third- or fourth-line drugs for such non-oncological indications. In this database study, we categorized these medications by their main indication into the oncological drug class, since this represented their by far most common use. For instance, the anti-CD20 monoclonal antibody rituximab was reimbursed in more than 90 % of all cases for patients with hematooncological indications, and only in a minority for patients with non-oncological indications, such as rheumatoid arthritis or immune thrombocytopenia. Thus, this misclassification is not expected to greatly affect our findings.

Practical solutions for reducing the costs of cancer therapies should consider different strategies beyond the well-established formulary approach, including implementation of pharmacoeconomic evaluation in health care decision-making. A number of strategies have been suggested, including sequential monotherapy use in the palliative setting (instead of combination therapy) and lowering the doses of anticancer agents when appropriate and supported by data [1]. Another well-known approach is the Technology Appraisal Program of NICE in the United Kingdom which often considers the cost per QALY ratio (quality adjusted life years) [14–16]. The measure of QALY is widely used as it takes into account not only life expectancy but also quality of life, which is often significantly impaired in cancer patients. Other potential solutions beyond cost sharing (i.e., increasing the contributions to insurance premiums and selection of insurance plans with higher copayment) include risk sharing [17] and conditional reimbursement [17, 18]. Risk sharing refers to financial compensation of health insurers by drug companies in cases where the post-marketing drug effectiveness was inferior to the benefits expected from the drug dossier submitted to the regulatory authorities for drug approval. Conditional reimbursement refers to temporary conditional approval of an index drug therapy for a limited period of time, after which its approval is reevaluated as a condition for permanent reimbursement.

## Conclusions

Our data suggest that despite successful MHS cost containment methods to control modern medication expenditure and thus provide updated treatment for chronic medical conditions to larger patient populations, the economic burden of cancer medications on the drug budget increases constantly, potentially compromising the total drug budget. Our findings suggest that a comprehensive cost-benefit assessment of cancer drug therapy should guide oncological drug approval in public health care organizations, and methods to control and limit drug cost should be coordinated between health care providers, pharmaceutical companies, and policy makers.

## Abbreviations

HMO: Healthcare maintenance organization
IQR: Inter-quartile range
MHS: Maccabi healthcare services
NIS: New Israeli Shekel
QALY: Quality adjusted life years
